# Standardized Localization of Intraoperative Direct Electrical Stimulation Sites in Awake Brain Surgery Using MRI-Based 3D Cortical Mapping

**DOI:** 10.3390/brainsci16101039

**Published:** 2026-09-29

**Authors:** Eleonora Grande, Andrea Bartolo, Chiara Rita Masotti, Alice Esposito, Vincenzo Esposito, Marco Ciavarro

**Affiliations:** 1 IRCCS Neuromed, 86077 Pozzilli, IS, Italyandreabartolo9@gmail.com (A.B.); marcociavarro@gmail.com (M.C.); 2 Department of Neuroscience, Biomedicine and Movement Sciences, University of Verona, 37129 Verona, VR, Italy; 3 Department of Human Neuroscience, Sapienza University of Rome, 00100 Roma, RM, Italy

**Keywords:** brain mapping, direct electrical stimulation, cortical mapping, brain functional localization

## Abstract

**Highlights:**

**What are the main findings?**

- An anatomically guided method enables precise localization of cortical intraoperative DES sites onto a brain atlas, enhancing the integration of functional mapping into research and clinical practice.

**What are the implications of the main findings?**

- Tested on 90 DES-identified eloquent sites, the approach yielded 87% inter-rater agreement with spatial discrepancies under 2.5 mm.
- The approach is broadly applicable, using open-access software and standard clinical neuroimaging, making it suitable for both clinical and research settings.

**Abstract:**

Objective: Intraoperative direct electrical stimulation (DES) remains the gold standard for mapping motor and cognitive cortical functions during awake brain surgery. However, a reproducible method to relocate these stimulation sites on the common brain template is still lacking, hindering reproducibility and data integration. Here, we propose our tested corticotopography method, as a reliable technique for mapping cortical DES sites onto brain atlases. Methods: Corticotopography was applied by matching the intraoperative image of the exposed cortex with the preoperative 3D MRI-based brain reconstruction using anatomical landmarks (i.e., sulci, gyri, and cortical vessels). Coordinates of the stimulation sites were then extracted using FSLeyes and mapped to MNI152 space. The method’s reliability was then evaluated through an inter-rater comparison. Lesion volume, tumor relapse status, and extent of craniotomy were tested for their potential bias on localization reliability. Results: Ninety eloquent cortical sites were identified in 19 patients undergoing awake surgery for glioma resection. Inter-rater analysis showed high concordance with 87% of stimulated sites presenting a spatial discrepancy below 2.5 mm. No significant differences in localization agreement were observed between newly diagnosed or relapsing tumors or as a function of lesion volume or craniotomy size. Conclusions: We introduce a standardized and reproducible corticotopography technique to localize intraoperative cortical stimulation sites on preoperative anatomical MRI. By formalizing the procedure used to map cortical functions, this method reduces inter-study variability, enabling DES to serve as a cumulative and interoperable resource for functional brain mapping. This allows cortical DES-derived information to be integrated with other neuroimaging modalities and leveraged across neuroscientific domains, including large-scale brain mapping and connectomics.

## 1. Introduction

The use of direct electrical stimulations (DES) under awake conditions has been an accurate intraoperative tool for the causal mapping of brain functions during brain tumor surgery for the last 100 years [1,2,3]. Although the mechanism underlying DES-induced effects is poorly understood [4], this technique is widely recognized as the gold standard for functional brain mapping [3,5,6]. DES enables causal investigation of brain–function relationships by transiently interfering with neural activity, producing reversible “virtual lesions” with measurable behavioral effects [7]. When applied during awake surgery, it enables the precise identification of functionally critical regions that must be preserved during resection. Also, since the early intraoperative stimulation studies by Penfield, DES has remained the more reliable source of causal evidence for neuroscience research [8,9]. In this scenario, the intraoperative region identification through DES still represents the best localization technique, with a spatial and temporal accuracy unmatched by any other available techniques [10,11]. Therefore, translating eloquent structures identified intraoperatively using DES to human brain atlases offers a unique way to investigate the functional organization of the human brain.

Several research groups have explored brain functioning by mapping DES-derived stimulation sites onto standardized brain templates [9,11,12,13,14,15]. However, the methodologies employed for localizing cortical stimulated points remain highly heterogenous and lack standardization. For instance, localization strategies have ranged from manual annotation of visually estimated points on schematic brain templates followed by digitization into the Montreal Neurological Institute 152 atlas (MNI152), to direct projection of individual intraoperative brain surfaces onto a standardized reference brain [11]. These approaches are inherently prone to spatial bias due to the difficulty of accurately matching the highly variable intraoperative brain morphology to standardized MNI space. Intraoperative neuronavigation systems have also been employed to localize cortical and subcortical eloquent areas [6,16,17], yet their precision is often undermined by intraoperative brain shift, a major source of localization error [18].

Taken together, this means a lack of methodological consistency among techniques, as well as a lack of reproducibility, ultimately undermining the scientific validity and generalizability of DES-based functional brain mapping.

In the literature, the need to overcome these limitations was recently highlighted in a study by Elia and colleagues [19], who highlighted the importance of accurately localized DES sites. They proposed a new localization method matching intraoperative brain photography with postoperative MRI. The coregistration of intraoperative photographs showing marked positive subcortical sites with the 3D reconstruction of postoperative MRI enables the identification of anatomical landmarks, such as the borders of the resection cavity, which facilitate accurate localization of stimulated subcortical sites on MRI and subsequent coordinate extraction. 

However, important information about brain function can also be obtained from cortical stimulation sites; therefore, it is also essential to localize these sites accurately.

In this case, postoperative MRI may not be directly comparable to the cortical surface exposed immediately after dural opening. Indeed, previous surgical procedures can induce anatomical distortions, leading to poor correspondence between the postoperative MRI surface and the exposed cortical surface on which brain mapping is performed before resection begins.

To overcome this limitation, we propose a new method for localizing intraoperative cortical stimulation sites, by directly mapping them onto preoperative anatomical MRI using a standardized procedure. Because the exposed cortical surface during surgery closely resembles the cortical surface reconstructed from preoperative MRI—unlike postoperative images, which are affected by surgically induced anatomical distortions—this approach enables more accurate spatial recognition of anatomical landmarks, meaning more accurate spatial recognition of cortical stimulation sites.

For this purpose, we integrated a previously proposed lesion-localization technique developed at our institution: MRI-based corticotopography [20]. This method relies on distinct morphological brain patterns, e.g., sulci, gyri, and cortical vessels, to precisely localize regions on the cortical surface reconstructed from preoperative structural MRI.

Originally developed for epilepsy surgery, it was subsequently extended to identify the cortical projection of deep-seated brain lesions, thereby assisting surgical trajectory planning when lesions are not directly visible on the cortical surface.

Specifically, this technique exploits the complementary information provided by multiple clinical 3D anatomical MRI sequences to identify recognizable cortical landmarks. Fluid-attenuated inversion recovery (FLAIR) images facilitate the visualization of cortical sulci and gyri, whereas cortical vessels are delineated on contrast-enhanced T1-weighted sequences.

Building on its procedural simplicity, we propose to extend this technique to the localization of cortical stimulation sites. This approach offers a practical and widely accessible tool to standardize the spatial representation of cortical DES mapping across patients. To this end, we implemented a double-blind site localization procedure and assessed its reliability through an inter-rater validation analysis.

By refining the localization of DES sites within a reproducible, MRI-based framework, this study aims to contribute to the standardization of intraoperative functional mapping. Ultimately, this standardization supports greater consistency in brain mapping data, enabling more reliable integration of DES findings into neuroimaging applicability and facilitating their translation into broader neuroscientific applications, from probabilistic atlases to connectomics application.

As a secondary aim, we investigated whether intrinsic lesion-related factors may influence the reliability of this mapping procedure. Lesion size may induce mass effects that reduce the visibility of cortical folding, while craniotomy extent directly affects the identification of cortical landmarks by limiting the exposed cortical surface. Additionally, in relapsing tumors, previous surgical procedures can induce anatomical distortion that further complicate landmark identification. To address these factors, we enrolled a heterogeneous cohort of patients undergoing surgery for first diagnosis or relapsing tumors, characterized by varying lesion volumes and craniotomy sizes, and evaluated whether these features were associated with inter-rater disagreement in site localization.

## 2. Materials and Methods

### 2.1. Patients Group

A consecutive series of patients who underwent glioma resection in “awake surgery” between 2021 to 2024 were included. Inclusion criteria were (1) age ≥ 18 years and signed informed consent; (2) diagnosis of supratentorial glioma according to the 2021 WHO classification of central nervous system tumors; (3) function-based resection with intraoperative functional brain mapping using DES and under awake condition; (4) availability of intraoperative recordings of surgical microscope video/photo documentation of stimulated sites.

All patients provided written informed consent before participation, including consent for the research use of clinical materials. The study protocol, including the secondary research processing of these materials, was approved by the Territorial Ethics Committee of ASReM. Before research processing, MRI data and intraoperative images were pseudonymized and assigned a study-specific identification code. The correspondence between study codes and patient identifiers was stored separately from the research dataset and was accessible only to authorized personnel. Pseudonymized imaging data were processed using FSL/FSLeyes version 1.15.0 [21] and the other imaging software described in the Methods within the institution’s secure data environment.

### 2.2. MRI Acquisition and MRI-Based Analysis

Preoperative MRI scans were acquired at the Neuroradiology Unit of IRCCS Neuromed (Pozzilli, Italy) using 3 Tesla GE Signa HDxT scanner (General Electric Medical Systems, Milwaukee, WI, USA). The protocol included the following: (1) a high-resolution structural 3D T1-weighted post-gadolinium image acquired using a spoiled gradient recalled echo (SPGR) sequence (TR = 10.26 ms, TE = 4.19 ms, slice thickness = 1 mm); (2) 3D T2-weighted FLAIR sequence (TR = 6000 ms, TE = 132.5 ms, slice thickness = 1.8 mm).

Lesion volumes were obtained by manual lesion segmentation by a senior neuroradiologist (AB) on the preoperative T1-post gadolinium and FLAIR images on 3D Slicer (version 5.8.1, www.slicer.org).

To estimate the surface area of the craniotomy, the postoperative CT images were acquired at 48 h post-surgery. CT images were imported into 3D Slicer and cranial bone was segmented using the Segment Editor followed by manual refinement to remove non-cranial structures by a senior neuroradiologist (AB). A three-dimensional surface model of the skull was generated. Then, the craniotomy margin was delineated directly on the three-dimensional skull surface using the built-in Markups module with the Closed Curve tool. This approach allowed the contour to conform to the native curvature of the cranial surface. The enclosed area was automatically computed by the software and expressed in square millimeters (cm^2^). Only surface-based measurements were performed; no volumetric calculations were included.

### 2.3. Intraoperative Stimulation Procedure

A monitored anesthesia care (MAC) protocol was adopted, allowing the patient to remain awake and responsive for intraoperative functional testing.

Preoperative 3D cortical surface reconstructions were generated from post-contrast T1-weighted and T2-weighted FLAIR images using BrainWavePA (GE Healthcare, Chicago, IL, USA, version 1.6.3), a software platform approved for clinical use and routinely employed for surgical planning in our institution. Following dural opening, these reconstructions were visually compared with the exposed brain surface. Corresponding patterns of cortical sulci, gyri, and vessels were used to recognize and delineate the exposed cortical region, localize the lesion relative to the cortical surface, and define the surgical trajectory (Figure 1A–C), according to the clinical corticotopography procedure described by Esposito et al. [20]. Once the lesion and surgical trajectory had been identified, the operative microscope was introduced into the surgical field, and video recording of the exposed cortex was initiated.

The intraoperative functional brain mapping was then performed by selectively stimulating the exposed cortical surface through DES. A bipolar electrode 5 mm inter-tip distance was employed, delivering biphasic current at 60 Hz 1 ms phase duration (Osiris NeuroStimulator, Inomed, Emmendingen, Germany).

During stimulation, patients performed several language tasks (i.e., serial order, object naming—DO-80 [22], verbs naming [23], repetition of sentences). A stimulation site was considered “positive” when language errors occurred during at least three non-consecutive stimulations. Language errors included any stimulation-induced disturbance affecting language production, such as anomia, phonemic or semantic paraphasia, pure speech arrest, and motor speech deficits including dysarthria. A senior neuropsychologist (MC) monitored the patients’ responses and reported any errors to the surgeon, who then marked the corresponding cortical site. The cognitive value of these errors is not the focus of this work and will not be discussed further.

Each positive site was labeled with a sterile, numbered 5 mm square tag. Microscope photographs documenting the exposed cortical surface and the labeled stimulation sites were acquired during cortical mapping and before corticotomy, tumor resection, or any substantial manipulation of the exposed cortex.

Once functional margins were identified; tumor resection was carried out under continuous behavioral monitoring.

### 2.4. Intraoperative Cortical Sites Localization

Although the BrainWavePA reconstructions enabled clinical corticotopographic identification of the exposed cortical region, the software does not provide a function for extracting the MRI coordinates of points identified on the reconstructed cortical surface. Therefore, a separate localization procedure was implemented in FSLeyes, version 1.15.0, included in the FSL software package [24], to localize the individual stimulation sites and extract their coordinates.

For this purpose, the native FLAIR images were processed using the brain extraction tool (BET) to remove non-brain tissues, including the skull, scalp, and dura. An initial fractional intensity threshold of 0.5 was applied and subsequently adjusted, when necessary, following visual inspection to optimize brain extraction. The skull-stripped FLAIR images were then rendered as three-dimensional cortical surfaces using the built-in FSLeyes (version 1.15.0) 3D viewer. FLAIR images were selected for this procedure because the post-contrast T1-weighted images frequently provided suboptimal cortical surface contrast in FSLeyes.

Each stimulation site was independently localized by two neurosurgeons (CRM and AE), who were blinded to each other’s localization results.

Within the FSLeyes environment, the raters visually matched the sulcal and gyral landmarks visible in the intraoperative photograph acquired before starting brain manipulation with those displayed on the FLAIR-derived cortical surface (Figure 1D).

When the surface contrast of the FSLeyes rendering was insufficient for unambiguous recognition of the exposed cortical region, the BrainWavePA reconstructions derived from the post-contrast T1-weighted and FLAIR images were consulted as additional visual anatomical references. The more clearly recognizable patterns of sulci, gyri, and cortical vessels displayed in BrainWavePA facilitated orientation and delineation of the exposed cortical region on the FSLeyes rendering, as indicated by the yellow outline in Figure 1D. This comparison was exclusively visual and did not involve spatial coregistration or coordinate transfer between the two software environments.

To examine whether the cortical landmarks required for this visual matching could also be identified using an open-source platform, we additionally generated a cortical surface reconstruction of the same patient shown in Figure 1 in the 3D Slicer package using the same BET procedure on T1-post gadolinium and T2 FLAIR. The relevant cortical landmarks were comparably recognizable, as shown in Supplementary Figure S1.

For each stimulation site, each expert extracts and stores the coordinates (x, y, z) extracted on FLAIR native space and visualized on FSLeyes (Figure 2). To transform each stimulation coordinate into an ROI, a custom a Python script was implemented (Python version 3.13.12. Input coordinates provided in anatomical space (millimeters) were converted into voxel indices by inverting the image’s affine transformation matrix. A binary three-dimensional mask was then generated, assigning a value of 1 to all voxels within a user-defined radius of 2.5 mm (considering 5 mm bipolar inter-tip stimulator dimension) from the center point. The resulting mask was saved in NIfTI format and aligned with the source image for subsequent visualization. This process yielded a 5 mm diameter ROI for each stimulation site. The same procedure was repeated for each positive site, identified by both neurosurgeons.

### 2.5. Intraoperative Sites Inter-Rater Procedure

To validate the corticotopography technique as a DES localization method, we compared the blind inter-rate localization performed by two expert neurosurgeons by calculating the spatial difference for each stimulation site on the patient’s native space. The Euclidean distance (d) between the two ROI coordinates (x1, x2; y1, y2; z1, z2), reconstructed for the same stimulation site on the FLAIR image, was computed. Coordinates from the scanner-native space (as reported by FSL/FSLeyes in continuous world coordinates, units = mm) were used to calculate the Euclidean distance between two points. A spatial difference > 2.5 mm between the ROIs was considered significant, being more than half of the bipolar stimulator inter-tip distance.

Then, the spatial differences were used to evaluate whether clinical features—i.e., first diagnosis vs. relapsing tumors, lesion volume, craniotomy extension—might influence localization reliability. For this purpose, a linear mixed-effects model was used to assess the relationship between inter-rater Euclidean distance and patient-level factors (e.g., surgery timing, tumor volume, craniotomy area). Euclidean distance was treated as the continuous outcome, with surgery timing, tumor volume and craniotomy area included as fixed effects. A random intercept for each patient accounted for multiple measurements per individual. Statistical analyses were implemented in the open-source statistical software JASP (https://jasp-stats.org). The significance threshold was set at *p* < 0.05.

### 2.6. Intraoperative Cortical Sites Normalization on Brain Atlas

To enable the use of stimulation points on reference brain template, the FLAIR images were normalized to MNI152 space using SPM12. Once the deformation field was obtained, it was applied to the coordinates of each cortical eloquent site, transforming their location from the native space to the standardized template space. Neuroimaging data normalization was performed using SPM12—Clinical Toolbox. This process allowed for the transformation of all stimulation site coordinates into standardized MNI152 space across the entire patient group.

## 3. Results

### 3.1. Patients Clinical Data

Nineteen patients (6F) satisfied our inclusion criteria. Six patients (31,5%) were undergoing surgery for glioma recurrence. The mean patient’s age was 39.4 ± 12.4 years. The histological diagnosis was the following: five (26.3%) glioblastoma IDH wild-type IV grade, three (15.8%) oligodendroglioma IDH mutant III grade WHO, three (15.8%) oligodendroglioma IDH mutant II grade WHO 3, seven (36.8%) astrocytoma IDH mutant, one (5.3%) ganglioglioma. Lesions were localized in the left frontal lobe in 10 cases (52.7%), in the left parietal lobe in five cases (26.3%), and in the left temporal lobe in four cases (21.1%).

Table 1 reports patient demographic and clinical data such as lesion localization, volume and craniotomy area.

### 3.2. Intraoperative Sites Identification

Brain mapping was performed and registered in the entire cohort of 19 patients, yielding a total of 90 reproducible sites, meaning those sites that induced language errors among three independent stimulations (Figure 3). Of these, 26 sites (28%) were recorded in patients treated for relapsing tumors. Figure 3 showed the 90 recorded stimulation sites as those identified by the first rater.

### 3.3. Intraoperative Sites Inter-Rater Validation

The inter-rater validation method showed good consistency, with a mean spatial distance of 1.16 (±1.1 mm) suggesting a good reliability of this localization method, and 78 (87%) stimulation points in which the Euclidean spatial difference was lower than 2.5 mm. In the other 12 (13%) small spatial differences emerged, all ranging under 5 mm (i.e., bipolar stimulator inter-tip distance).

As secondary aim was to test whether the lesion and surgical features would affect the corticotopography localization method.

Linear mixed-effects models were separately performed to investigate whether surgical timing, craniotomy size, and lesion volume influenced the Euclidean distance between stimulation site localizations performed by the two raters. In all analyses, subject identity was included as a random intercept to account for repeated measurements within patients.

The first model assessed the effect of surgical timing (first, second, or third surgery) on localization discrepancy and revealed no significant effect $\left(F(2,14.40)=1.06,\;p=0.372\right)$, suggesting that repeated surgeries or tumor recurrence did not significantly affect inter-rater agreement.

A second model evaluated the effect of craniotomy size on Euclidean distance. No significant association was observed $\left(F(1,13.13)=1.95,\;p=0.186\right)$, indicating that localization agreement was not significantly influenced by the extent of cortical exposure. Finally, a third model tested the effect of lesion volume on localization discrepancy and similarly showed no significant effect $\left(F(1,11.59)=0.18,\;p=0.682\right)$. This finding suggests that the proposed corticotopography-based localization method remains reliable even in the presence of large lesions and potential mass effects altering cortical anatomy.

Overall, these analyses support the robustness of the proposed localization approach across different surgical conditions and anatomical scenarios.

## 4. Discussion

Accurate localization of brain functions is essential for understanding the intrinsic organization of the human brain. To this purpose, the DES remains the most important tool to directly interrogate the brain [25]. DES transiently disrupts neuronal activity through mild electrical stimulation, enabling identification of eloquent areas during brain tumor surgery and the causal mapping of brain function [3,8,26].

Nevertheless, to extract information on the deep organization of the brain, brain mapping data from intraoperative stimulation must be moved from the intraoperative space and transferred onto reference brain atlases. To date, a standardized methodology for this process is lacking. This substantially reduces the generalizability and scientific potential of data derived from intraoperative functional mapping.

To overcome this limitation, the present work proposes and validated a novel localization method to accurately transfer cortical stimulation sites onto a reference brain atlas. We employed corticotopography, a technique originally developed for localizing subdural grid electrodes and later adapted to guide surgical trajectories for deep-seated lesions. This approach relies on visual matching between three-dimensional preoperative MRI reconstructions and the intraoperative cortical surface by identifying characteristic patterns of sulci, gyri, and cortical vessels used as anatomical landmarks. These landmarks remain relatively unaffected by brain shift, providing a reliable reference for cortical localization [20,27].

This gave us the impetus to propose this method for the localization of cortical stimulation sites recorded during surgery. Intraoperative stimulation sites were matched with 3D FLAIR reconstructions using cortical landmarks (sulci and gyri), allowing extraction of MRI coordinates of stimulated points and their transformation into standard space. When needed, clinically derived post-contrast T1 image reconstruction obtained in BrainWavePA improved vessel-based matching. Then, to assess the reliability of this procedure, a double-blind inter-rater validation was performed here. Eighty-seven percent of stimulation sites showed concordance between the two independent localizations, supporting the reliability of this method for transferring cortical stimulation sites into MRI coordinates.

As a secondary aim, we investigated whether surgical and lesion-related characteristics influenced localization reliability.

To further assess the robustness of the proposed localization method, we enrolled a heterogeneous cohort of glioma patients differing in surgical timing, lesion volume, and craniotomy size. Notably, none of these variables significantly affected localization agreement. In particular, the absence of differences related to surgical timing suggests that the method remains reliable even in recurrent tumors, where anatomical distortions are typically more pronounced due to previous resections and may affect the ability to recognize anatomical landmarks on 3D MRI reconstructions. Similarly, lesion volume did not influence localization agreement, indicating that the method is resilient even in the presence of marked mass effect, which could worsen the visualization of cortical folding on 3D reconstructions. Craniotomy size also showed no significant effect, supporting the applicability of corticotopography even in less extensive surgical exposures, without substantially impairing the identification of cortical landmarks on the exposed cortical surface. However, since the smallest craniotomy in our cohort measured 15.24 cm^2^, we cannot exclude that more limited cortical exposure may reduce the visibility of anatomical landmarks and consequently affect localization reliability.

These findings support the idea that matching intraoperative views with MRI-derived brain surface reconstructions through the identification of anatomical landmark patterns represents a valuable tool for DES localization, enabling greater standardization in brain mapping independently of lesion-related clinical features. Moreover, this approach may help overcome several current limitations in the field, including inadequate control of brain shift in neuronavigation-based procedures [6,16,17] or reduced anatomical fidelity in methods relying on intermediate template transformations prior to normalization [11].

This limitation has recently been addressed by Ng S and colleagues [15]. They proposed to use postoperative MRI brain surface for comparison with intraoperative photographs. This allows a 3D visualization of individual cortical structures and a semi-automated MNI coordinate extraction. Although showing good reliability, this procedure does not clearly describe how coordinates are transformed from patient native-space to standardized MNI space.

Similarly, Elia et al. [19] focused on subcortical stimulation sites and used postoperative MRI as a reference for localization. This work clearly describes the coordinates transformation procedure from patient’s space to standardized brain space. Moreover, the authors assessed the anatomical and functional plausibility of the resulting localizations by comparing the subcortical sites with tractography atlas, showing that most sites intersected a white matter tract, consistent with the clinical response elicited during stimulation. Their findings therefore support the broader feasibility of combining intraoperative photographs with patient-specific MRI to localize DES-derived eloquent sites. However, their workflow was specifically designed for subcortical sites, which need postoperative MRI to be localized. Nevertheless, the postoperative MRI may be less accurate if employed for cortical stimulation site localization. Indeed, the postoperative brain anatomy can be affected by brain shift due to cerebrospinal fluid loss or postoperative brain depletion, and, more generally, by surgical-induced anatomical distortion. Thus, to prioritize accurate cortical localization, we relied on preoperative MRI sequences, which most closely resemble the intraoperative anatomy of the cortex soon after dura opening.

Overall, the general principle of corticotopography—matching intraoperative views with patient-specific MRI-derived representations—has been applied in different ways according to the anatomical target and clinical purpose. To our knowledge, the present study provides the first systematic assessment of inter-rater reliability for a corticotopography-based workflow that uses preoperative MRI to localize cortical DES sites and extract their coordinates. Results show that this technique proves to be a reproducible procedure, easy to apply in the clinical setting—requiring the use of a 3D reconstruction clinical MRI and intraoperative scene images—helpful to reach a standardization in the DES localization method, and without risk of bias for intrinsic clinical conditions.

This may represent a way to address the preliminary methodological requirement for subsequent multimodal applications: the reliable transfer of cortical DES sites from the intraoperative field to patient-specific MRI and standardized brain space. Once this localization step has been established, future studies may compare the resulting sites with different neuroimaging analysis (e.g., task-based fMRI, connectome analysis) as well as with different intraoperative observations (i.e., positive sites, negative sites).

Several limitations of this study should be acknowledged. First, the quality of cortical surface reconstruction and landmark identification depends on the spatial resolution and contrast of the preoperative MRI. In particular, acquisitions performed at lower magnetic field strengths (e.g., 1.5 T) may provide reduced tissue contrast and spatial detail, potentially limiting the reliability of surface-based matching and cortical site localization. In addition, the reliability of corticotopography may partially depend on the operator’s familiarity and experience with the technique, particularly in recognizing anatomical landmark patterns across intraoperative views and MRI reconstructions. Therefore, future studies should assess the reproducibility and inter-center reliability of this approach in independent clinical settings.

Second, our workflow relied in part on FSLeyes, an open-source software package, for 3D visualization of MRI data. While FSLeyes offers flexibility and accessibility, it does not always provide optimal surface rendering quality, especially for post-gadolinium T1-weighted images, where cortical and vascular contrast may be suboptimal. To address this limitation, we integrated BrainWavePA in some cases, a commercial software platform, which enabled more reliable surface reconstruction and anatomical landmark identification within a clinical approved software package. BrainWavePA was selected for this purpose because these reconstructions had already been generated and used intraoperatively for lesion localization and surgical planning. However, the use of commercial software may limit the immediate reproducibility of this approach in centers without access to such tools. Importantly, previous study [20] demonstrated that alternative open-source software solutions can achieve high-quality cortical surface visualization, including post-gadolinium images (i.e., MRIcro software package). In the present study, we directly verified the feasibility to obtain the 3D brain cortical surface using the 3D Slicer package. The resulting reconstruction provided a visualization of the sulcal and gyral anatomy comparable to BrainWavePA (Supplementary Figure S1), leveraging the generalizability of this procedure.

Finally, we implemented the localization procedure on T2-weighted FLAIR images because the three-dimensional surface reconstruction of post-contrast T1-weighted images in FSLeyes frequently provided suboptimal cortical surface delineation. Although alternative open-source platforms, such as 3D Slicer, may provide better visualization of post-gadolinium T1-weighted cortical surfaces (Supplementary Figure S1) with good vessel representation, the standard graphical interface does not directly display the spatial coordinates corresponding to the crosshair intersection within the three-dimensional view. Implementing this function would require dedicated scripting, thereby reducing the simplicity and accessibility of the proposed workflow for users without programming expertise. Future prospective studies could develop and systematically assess a unified open-source workflow combining high-quality cortical and vascular surface visualization with direct coordinate extraction. In particular, the integration of dedicated vascular imaging, such as MR venography, could enable cortical venous anatomy to be incorporated more systematically and potentially improve landmark recognition when sulcal and gyral morphology is distorted by the lesion.

## 5. Conclusions

In conclusion, the proposed corticotopography technique provides a reliable and practical approach for localizing intraoperative cortical stimulation sites on preoperative anatomical MRI. By formalizing the workflow for cortical functional mapping, this method moves DES beyond a case-specific intraoperative tool toward a cumulative and interoperable source of information on brain functions, while remaining robust to potential bias related to lesion volume, craniotomy extent, and tumor recurrence.

Atlas-consistent localization further enables the integration of DES-derived data with other neuroimaging modalities. This approach allows DES data to be used in future studies across several neuroscientific fields, including large-scale functional brain mapping and connectomics.

## Figures and Tables

**Figure 1:**
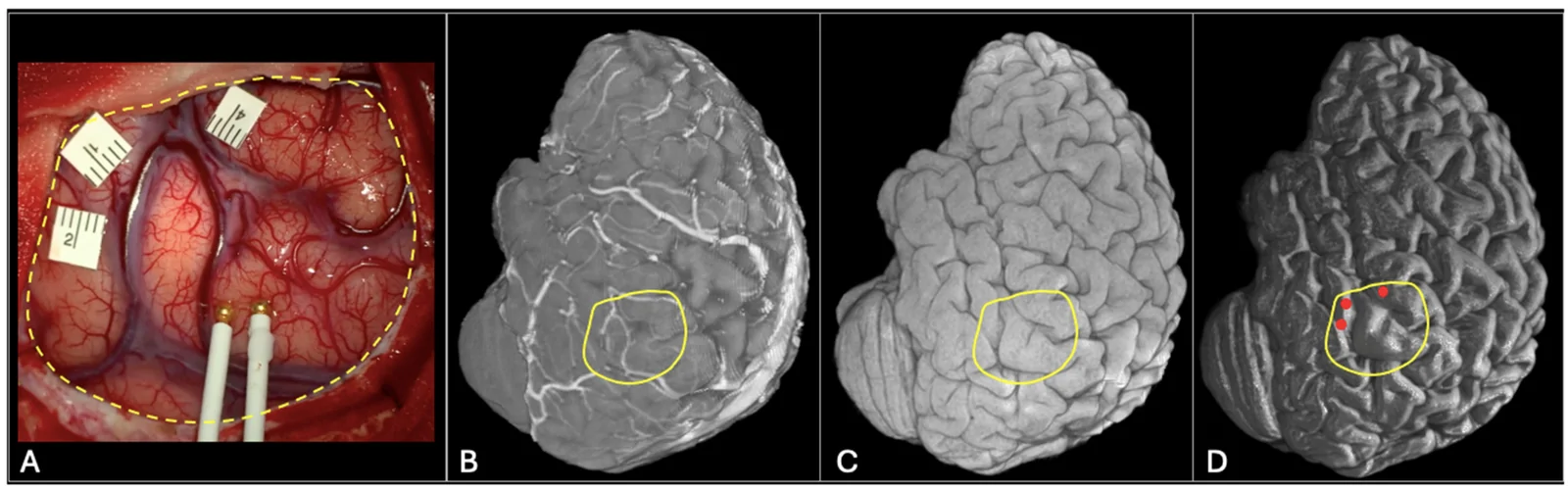
Stimulation site localization on 3D MRI reconstruction using intraoperative brain surface matching through sulci, gyri, and vessels. (**A**) Intraoperative recording of exposed brain surface and positive sites; (**B**) BrainWavePA T1 post-gadolinium 3D brain surface reconstruction for intraoperative matching; (**C**) BrainWavePA T2 Flair 3D brain surface reconstruction for intraoperative matching; (**D**) FSLeyes T2 Flair brain surface reconstruction for stimulation site identification. Yellow lines show the MRI design of exposed cortex; red dots represent positive stimulated site localization.

**Figure 2:**
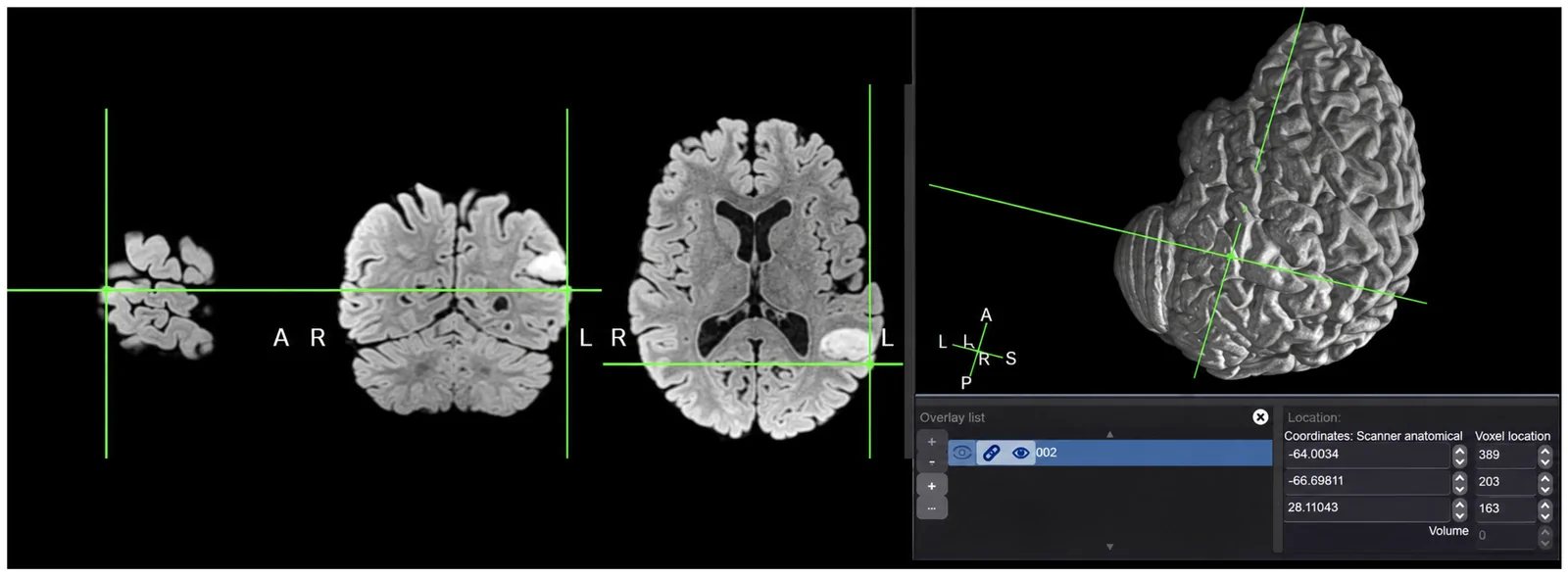
Coordinate identification for each stimulation site in native-scanner space (mm).

**Figure 3:**
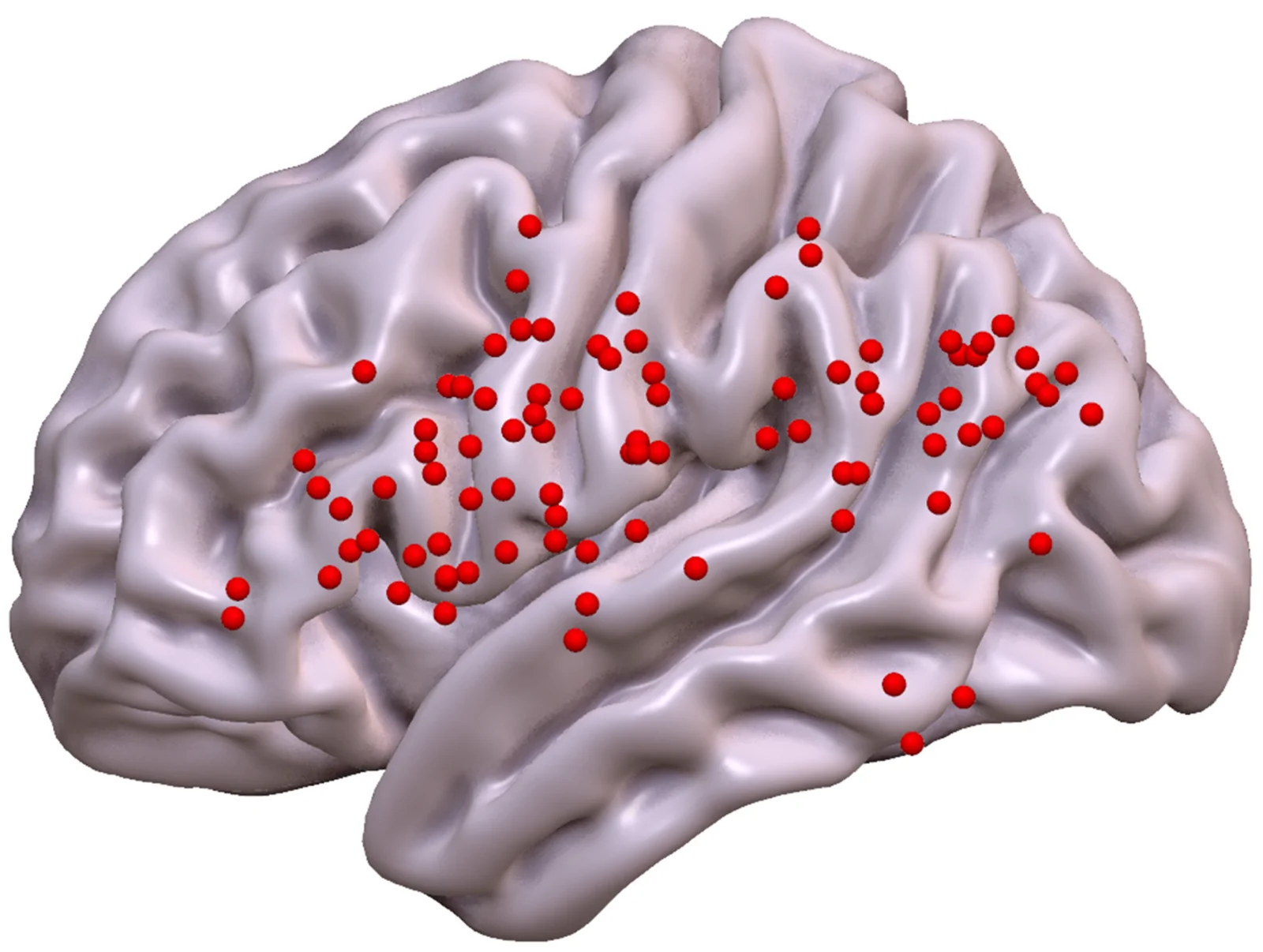
Red dots show positive stimulation sites eliciting intraoperative deficits.

**Table 1:** Patients’ demographic, clinical and lesion data.

Demographic		Clinical Data					
Gender	Age	Istology	Tumor Grading (WHO2021)	Lesion Localization	Surgery	Lesion Volume	Craniotomy Area
M	31	Oligodendroglioma IDH mutant	III	Frontal	First	104	33.9
M	45	GBM IDH wildtype	IV	Parietal	First	18.7	24.87
M	19	Astrocytoma IDH mutant	II	Parietal	First	8.47	26.58
F	39	Oligodendroglioma IDH mutant	II	Frontal	Third	7.29	35.51
M	29	Oligodendroglioma IDH mutant	II	Frontal	First	29.6	15.24
F	48	Astrocytoma IDH mutant	II	Frontal	Second	11.2	43.91
M	60	GBM IDH wildtype	IV	Frontal	First	8.19	26.05
M	32	Oligodendroglioma IDH mutant	II	Temporal	First	18.8	27.02
M	30	Astrocytoma IDH mutant	II	Frontal	First	23.2	26.56
M	64	GBM IDH wildtype	IV	Temporal	First	18.5	46.9
F	38	Astrocytoma IDH mutant	II	Frontal	First	11.3	43.56
F	58	GBM IDH wildtype	IV	Temporal	First	11.1	29.45
F	29	Astrocytoma IDH mutant	II	Frontal	Third	5.08	32.81
M	34	GBM IDH wildtype	IV	Parietal	First	11	29.02
M	48	Oligodendroglioma IDH mutant	III	Parietal	Second	17.1	35.42
M	43	Astrocytoma IDH mutant	III	Frontal	First	8.78	51.18
M	44	Ganglioglioma	I	Temporal	First	1.2	18.84
F	38	Oligodendroglioma IDH mutant	III	Frontal	First	6.24	30.66
M	21	Astrocytoma IDH mutant	II	Parietal	Second	13.3	26.36

## Data Availability

The original contributions presented in this study are included in the article. Further inquiries can be directed to the corresponding author.

## Supplementary Materials

The following supporting information can be downloaded at: https://www.mdpi.com/article/10.3390/brainsci16101039/s1. Figure S1: 3D brain surface reconstruction. Stimulation sites localization on 3D MRI reconstruction using intraoperative brain surface matching through sulci, gyri and vessels. A-D boxes represented the reconstruction already presented in the main text. Panels E1 and E2 show T1 post-gadolinium and T2 FLAIR views of the cortical surface reconstruction generated from the same patient using the open-source 3D Slicer platform. The relevant sulcal and gyral landmarks were comparably recognizable, supporting the feasibility of using 3D Slicer for the anatomical landmark-identification step.
